# Ultra-high pressure improved gelation and digestive properties of Tai Lake whitebait myofibrillar protein

**DOI:** 10.1016/j.fochx.2023.101061

**Published:** 2023-12-09

**Authors:** Mingfeng Xu, Xiangxiang Ni, Qiwei Liu, Chengcheng Chen, Xiaohong Deng, Xiu Wang, Rongrong Yu

**Affiliations:** a College of Life and Environmental Sciences, Hangzhou Normal University, Hangzhou 311121, China; b School of Advanced Materials & Engineering, Jiaxing Nanhu University, Jiaxing 314001, China; c The First Affiliated Hospital of Wenzhou Medical University, Wenzhou 325000, China

**Keywords:** Ultra-high pressure, Physicochemical properties, Gelling properties, *In vitro* digestion characteristics, Tai Lake whitebait, Myofibrillar protein

## Abstract

This study investigated the effects of ultra-high pressure (UHP) at different levels on the physicochemical properties, gelling properties, and *in vitro* digestion characteristics of myofibrillar protein (MP) in Tai Lake whitebait. The α-helix gradually unfolded and transformed into β-sheet as the pressure increased from 0 to 400 MPa. In addition, the elastic modulus (G') and viscous modulus (G'') of the 400 MPa-treated MP samples increased by 4.8 and 3.8 times, respectively, compared with the control group. The gel properties of the MP also increased significantly after UHP treatment, e.g., the gel strength increased by a 4.8-fold when the pressure reached 400 Mpa, compared with the control group. The results of *in vitro* simulated digestion showed that the 400 MPa-treated MP gel samples showed a 1.8-fold increase in digestibility and a 69.6 % decrease in digestible particle size compared with the control group.

## Highlights

• Ultra-high pressure improved the emulsification performance of whitebait MP.
• Ultra-high pressure induced molecular stretching of whitebait MP.
• Ultra-high pressure enhanced the gel strength of whitebait MP.
• Ultra-high pressure improved the digestibility of whitebait MP gel.

## 1 Introduction

Tai Lake whitebait is a type of fish that falls under the order *Salmoniformes* and *Scleractinia*. This fish is known for its high protein content and low-fat composition, making it a nutritious choice. According to China Fishery Statistical Yearbook 2023, the output of Lake whitebait in China reached 12,177 tons in 2022. The main product forms of Lake whitebait include soft-packed ready-to-eat leisure food, dried products, puffed food, etc. The production scale of whitebait-related products is on the rise year by year (Liu, 2023). Currently, whitebait products on the market are mostly processed in the form of whole fish for consumption, with fewer refined and deep-processed products. More than 50 % of the muscle protein composition in Lake whitebait is myofibrillar protein (MP), which has good gelatinizing ability and is the main component of surimi products (Deng et al., 2023; Li et al., 2022a). In general, MP is primarily composed of myosin, actin, actinomycin, and troponin. Its biochemical characteristics are linked to the properties of emulsification, tissues, rheology, gelation, and flavor in aquatic muscle profucts. The superiority of surimi products is greatly influenced by the gelatinization of MP. The functional properties of proteins are closely connected to the structure of MP, which subsequently impacts the quality of surimi products. Consequently, comprehensively investigating the conformational and functional aspects of MP will contribute to the production of cost-effective, nourishing, and wholesome processed aquatic foods (Kim et al., 2012, Li et al., 2022).

The nutritional and functional properties of proteins may not always be optimal, prompting scholars to explore physical, chemical, and enzymatic alterations to enhance these characteristics. However, certain chemical modification techniques such as acid-base and glycosylation are now employed sparingly due to concerns about food safety and environmental pollution. Therefore, further research is necessary to investigate enzyme modification methods, considering factors such as production cost, selection of enzyme sources, and modification efficacy (Zink, Wyrobnik, Prinz, & Schmid, 2016). Emerging physical technologies such as low-temperature plasma, ultra-high pressure (UHP), and ultrasound have demonstrated their superiority in enhancing the modification of proteins, extending the shelf life of food, and improving the quality of products. These techniques offer high efficiency, minimal energy consumption, and a safe approach. Moreover, these methods facilitate the improvement of several physicochemical properties, such as solubility, hydrophobicity, and sulfhydryl content. An example of such a method is UHP, which is widely acknowledged as a traditional non-thermal physical processing technique. It has been successfully applied in fish processing as an innovative food processing technology (Wu et al., 2020, Zhang et al., 2024). The molecular conformation of proteins can be altered by it, leading to the enhancement of the exposure of reactive groups (e.g., hydrophobic groups and sulfhydryl groups) within the MP. This process also influences the solubility of the MP, resulting in a substantial impact on the gel properties of MP. Through experimentation, it was shown that subjecting crayfish MP to a UHP intensity of 300 MPa could modify the protein structure and effectively enhance the distribution of water (Huang et al., 2020, Shi et al., 2020). According to the findings of Liu et al. (2022), the application of a 200 MPa treatment significantly improved the solubility and digestibility of the mantle protein (MP) derived from scallops. Previous studies have shown that UHP treatments can influence the biochemical characteristics and alter the secondary structure of MP, while maintaining the nutritional value and taste of the associated food product. When a UHP treatment of 500 MPa was applied, it led to increased solubility and modifications in the secondary structure in a protein extracted from oysters. Additionally, the digestion process in the stomach showed a 50.19 % improvement in digestibility for the samples treated with 500 MPa, and a 34.78 % overall increase compared to the untreated sample (Wang et al., 2019a). Despite extensive research on the impact of UHP treatment on the structural and functional characteristics of meat protein, limited knowledge exists regarding its application to Tai Lake whitebait's MP.

The main objective of this research was to investigate the impact of UHP on the physicochemical properties, gelling properties, and *in vitro* digestibility of Tai Lake whitebait MP. To achieve this, the MP of Tai Lake whitebait was subjected to various UHP treatments (100, 200, 300, and 400 MPa). These treatments were used to assess their effect on carbonyl content, emulsification properties, and the secondary and tertiary structure of the MP. Additionally, the rheological properties, gel strength, and microstructure of the MP gel were evaluated to gain insights into the influence of UHP on the overall gel characteristics. Furthermore, the impact of UHP induced structural changes in the MP on the *in vitro* digestion of the MP gel was also determined. This study provides valuable information on the potential use of UHP to enhance the quality of surimi products and expands the application of UHP in the food industry.

## 2 Materials and methods

### 2.1 Materials and reagents

In October 2022, frozen whitebaits were purchased from Changxing Xintang Fishery Market (Changxing, China). The whitebaits had an average weight of 8.87 ± 0.41 g and measured approximately 9.47 ± 0.35 cm. The following chemicals used in the study were obtained from Sigma-Aldrich (St. Louis, MO, USA): 5,5′-Dithiobis-(2-nitrobenzoic acid) (DTNB), deloitte touche tohmatsu (DTT), ethylenediaminetetraacetic acid (EDTA), and trichloroacetic acid (TCA). Pepsin and trypsin were purchased from Yunrui Reagent Co., Ltd. (Hangzhou, China). All other chemicals used in this study were acquired from Qihao Chemical Scientific Co., Ltd. (Hangzhou, China) and were of analytical grade. The whitebait meat were utilized, stored at −30℃, and utilized within 48 h.

### 2.2 Extraction of MP from Tai Lake whitebait

The extraction method for Tai Lake whitebait MP was conducted according to the protocol described by Li et al. (2022). Briefly, 300 g of whitebait tail meat was minced and homogenized with four times the volume of phosphate buffer (100 mM Na_2_HPO_4_/NaH_2_PO_4_, 100 mM NaCl, 2 mM MgCl_2_, 0.001 M EDTA, pH 7.0) using a homogenizer (T25, IKA Corporation, Germany) at a speed of 10,000 rpm for 1 min. The resulting mixture was then centrifuged at 10,000 × g at 4℃ for 10 min. The collected precipitate was washed twice with phosphate buffer. This precipitate represents the extracted MP of whitebait and was stored at 4℃ for further use within 24 h.

### 2.3 UHP treatment of MP

The phosphate buffer was used to prepare a solution of extracted MP at a concentration of 50 mg/mL. This solution was then packed into polyethylene bags measuring 120 mm in length and 35 mm in width, with a volume of 30 mL per bag. To ensure freshness, the bags of each treatment fraction were divided into four and vacuum sealed. Additionally, a larger polyethylene bag filled with pre-cooled water was prepared. The water was carefully de-aerated, and the bag was tightly sealed before being used for the UHP process. Following the method described by Chen et al. (2014), the MP was treated using UHP equipment (L2-600/1, Huataisen Miao Biological Engineering Technology Co. LTD, Tianjin, China). The MP was treated separately with pressures of 100, 200, 300, and 400 MPa for 10 min at a temperature of 25℃. As a control group, a sample without UHP treatment was kept at 25℃ for 10 min. The samples, both before and after UHP treatment, were stored at a temperature of 4℃ and not kept for more than 5 h before further analysis.

### 2.4 Physicochemical properties of MP

#### 2.4.1 Carbonyl

The carbonyl content in MP samples was determined using the reagent test kit (No. A087-1–1, JIANCHENG Biology Co., ltd., Jiangning, China). The procedure followed the method described by Li et al. (2022a). The quantification of carbonyl content was reported as nmol/mg protein.

#### 2.4.2 Emulsifying activity index (EAI) and emulsion stability index (ESI)

To evaluate the EAI and ESI values, we followed a modified approach based on the study conducted by Li et al. (2022a). The experiment began by homogenizing the MP solution (5 mg/mL) with soybean oil (1:4, w/v) using an IKA Corporation blender (T25, Germany) operating at a speed of 10,000 r/min for 1 min. Then, we extracted 20 μL of this emulsion from the bottom and adjusted it to a volume of 5 mL by adding 1 mg/mL sodium dodecyl sulfate (SDS) to ensure thorough mixing. After that, the absorbance at 500 nm was measured using a TECAN microplate reader (Infinite M200, Switzerland), and this step was repeated every 10 min. The ESI and EAI were calculated by the following equation:

(1) $$EAI\left(m^{2}/g\right)=115.15\times A_{0}$$

(2) $$ESI\left(\%\right)=10\times A_{0}/\left(A_{0}-A_{10}\right)$$

Where A_0_ and A_10_ represent the absorbance values of emulsions at 0 and 10 min after homogenization, respectively.

#### 2.4.3 Total sulfhydryl groups

The method used to determine the overall levels of sulfhydryl was adapted from a previous study with minor adjustments (Li et al., 2021). Initially, 1.5 mL of MP (5 mg/mL) was combined with 9.5 mL of a solution containing 8 mol/L urea and 10 mmol/L EDTA (pH 6.0). Subsequently, 100 µL of Ellman's reagent (10 mmol/L DTNB in a 0.1 mol/L Na_2_HPO_4_/NaH_2_PO_4_ buffer) was added. The mixture was then placed in a dark environment at room temperature for 30 min, after which the absorbance at 412 nm was measured. As a control, supernatants without DTNB were used. The total sulfhydryl levels were determined using the following equation:

(3) Totalsulfhydrylcontent(μmol/gprot)=A×14.706

#### 2.4.4 Surface hydrophobicity (H_0_)

To assess the H_0_, we followed the approach described by Li et al. (2021). Initially, 1 mL of MP solution (2 mg/mL) was mixed with 200 uL of bromophenol blue (BPB) solution. The resulting mixture was then centrifuged at 5000 × g for 15 min at 4℃, and the precipitate was removed. For the control group, MP was not included in the solution. The absorbance was measured at 595 nm using a microplate reader. Finally, the calculation of H_0_ was performed using the formula provided below:

(4) $$H_{0}\left(\mu g\right)=200\mu g\times \left(A_{0}-A_{S}\right)/A_{0}$$

Where A_0_ and A_S_ represent the absorbance values of the control and samples, correspondingly.

#### 2.4.5 Intrinsic fluorescence spectroscopy

The intrinsic fluorescence spectroscopy of MP solution (0.2 mg/mL) was measured using a fluorescence spectrophotometer (Model RF-6000, SHIMADZU Co., Japan) (Li et al., 2021). The excitation wavelength was set to 280 nm, and the emission was measured in the range of 300–400 nm.

#### 2.4.6 Circular dichroism spectroscopy

The secondary structures of MP were measured using a J-1500 circular dichroism (CD) spectrometer (JASCO, Tokyo, Japan) (Deng et al., 2023). The concentration of the MP solution was adjusted to 0.2 mg/mL, and the data acquisition range was set at 190–260 nm.

#### 2.4.7 Rheological properties

The static rheological properties of MP were determined using a DHR-2 rheometer (TA Instrument, New Castle, UK) (Zhang et al., 2023). Parallel plates of 40 mm and 20 mm diameter were used. Oscillatory scans were performed in the frequency range of 0.1 to 10 Hz, and the temperature was set at 25℃. The relationship between the frequency scan and the elastic modulus (G') and viscous modulus (G“) of the Tai Lake whitebait MP was determined.

### 2.5 Gelling properties of MP gel

#### 2.5.1 Preparation of MP gel

The MP gels were prepared following the methodology described by Li et al. (2022a). Initially, a small beaker with a volume of 20 mL was filled with the MP solution. The sample height was maintained at 20 mm, and the opening was securely covered with plastic wrap. Subsequently, the sample was subjected to an initial heating phase at 40 °C for 60 min using a water bath, followed by a second heating phase at 90 °C for 30 min. After completion, the samples were rapidly cooled using pre-chilled water and stored at a temperature of 4 °C for approximately 12 h.

#### 2.5.2 Water holding capacity (WHC)

According to a study conducted by Li et al. (2022b), the centrifugation process was performed on 3 g of MP gel at a spped of 10000 × g for 15 min at 4℃. Afterward, the supernatant was removed, and the weight of the centrifuge tube and gel was measured before and after centrifugation. The WHC was determined using the following formula:

(5) $$WHC=\frac{W_{2}}{W_{1}}\times 100\%$$

where W_2_ is the total weight (g) of the centrifuge tube and the gel after centrifugation; W_1_ is the total weight (g) of the centrifuge tube and the gel before centrifugation.

#### 2.5.3 Gel strength

The strength of MP gel was evaluated using a texture analyzer (TA.XT. Plus, Stable Micro Systems, UK) equipped with a P/0.5 probe (Li et al., 2022a). The evaluation employed the following parameters: pre-test speed of 5.0 mm/s, test speed of 1.0 mm/s, post-test speed of 5.0 mm/s, and trigger force set at 5 g. Gel strength was quantified by multiplying the breaking strength with the depth of the sag.

#### 2.5.4 Microstructure

Microstructural images were obtained using a cold-field scanning electron microscope (SEM, SU80 10, HITACHI, Japan) (Li et al., 2021). The preparation process involved immersing the sample containing the specimens in liquid nitrogen to lower their temperature to −180 °C. The stage was then placed in a vacuum chamber to eliminate moisture. Sublimation of the samples was carried out at −90 °C for 30 min to ensure complete removal of any remaining moisture. Gold spraying was applied to the samples, which were then visualized using an accelerating voltage of 2 kV.

### 2.6 In vitro digestion of MP gel

#### 2.6.1 Standardized static *in vitro* simulation of gastrointestinal digestion

The technique of *in vitro* digestion was evaluated using a slightly modified approach (Khulal, Ghnimi, Stevanovic, Rajkovic, & Velickovic, 2021). In summary, a triangular flask was used to combine 1.5 g of MP gel sample, 113 mL of a 0.1 M HCl solution, and 15 mg of pepsin. The resulting mixture was then subjected to a simulated digestion process in the stomach at a constant temperature of 37 °C for 2 h. Afterward, the pH of the digested solution was adjusted to 7.5. Subsequently, a composite system comprising of 56 mL of phosphate buffer, 30 mg of trypsin, 7.5 mM CaCl_2_, and 0.01 % NaN_3_ was added to the sample. The simulated digestion in the small intestine was conducted at a consistent temperature of 37 °C for a duration of 2 h.

#### 2.6.2 Digestibility

The protein content in the undigested MP gels was assessed using the Komas Brilliant Blue technique as described by (Monsoor and Yusuf, 2002). After the two-step enzymatic digestion process mentioned earlier (simulating gastrointestinal digestion *in vitro*), a small amount of the MP gel samples remained undigested in the mixture. To separate the undigested MP gel, 30 % TCA was added to the mixture, and the precipitate was separated using ashless filter paper. In the control experiment, 1.5 mL of evaporated hall water was used instead of the MP gel. Digestibility was calculated using the following formula:

(6) $$Digestibility=\frac{A_{1}-A_{2}}{A_{1}}\times 100\%$$

where A_1_ is the crude protein content of the sample; A_2_ is the crude protein content in filter residue after pepsin-trypsin digestion.

#### 2.6.3 Particle size after *in vitro* digestion

The size of the MP gel particles after digestion was analyzed using a laser particle size analyzer (DT-1202, Dispersion Technology., DTI, America) (Carbonaro, Maselli, & Nucara, 2015). The samples were diluted to a concentration of 1 %, and the particle size measurement was conducted within the range of 2 to 3000 nm.

### 2.7 Statistical analysis

At least three independent experiments were conducted to obtain replicates of all the parameters' measurements. To compare the results of the study, a one-way analysis of variance (ANOVA) and Duncan's multiple comparison were performed using IBM Inc.'s SPSS 26.0 software (Chicago, USA). Statistical significance was determined with a probability level of *p* < 0.05. The graphs in the document were generated using OriginLab Inc.'s Origin 2018 software (Northampton, USA) and Microsoft's PowerPoint 2019 (Seattle, USA).

## 3 Results and discussion

### 3.1 Carbonyl

**Fig. 1:**
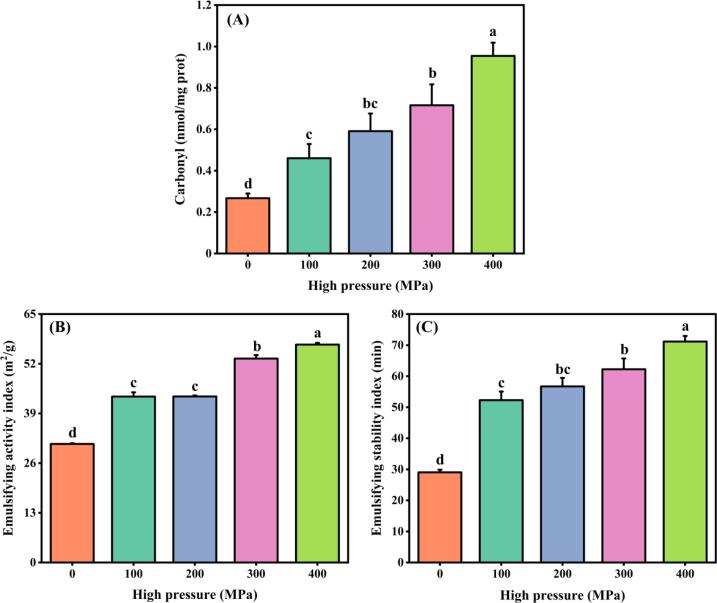
Carbonyl content (A), emulsifying activity index (B), and emulsifying stability index (C) of MP samples after UHP treatment. Different lowercase letters on each column represent statistically significant differences (*p* < 0.05).

The extent of protein oxidation can be determined by analyzing the carbonyl group (Shi et al., 2020). As depicted in Fig. 1A, compared with the control sample (0.27 nmog/mg), the carbonyl content of MP showed a significant increase with the gradual increase of UHP pressure. As an example, the carbonyl content of MP treated with 400 MPa increased 2.52-fold compared to the control. The application of gradually increasing pressure in the UHP process results in the compression of the protein system, leading to the formation of larger insoluble protein aggregates. This process has an impact on the structure of the proteins, which subsequently triggers the production of reactive oxygen and non-oxygen radicals by oxidizing enzymes (Bak, Bolumar, Karlsson, Lindahl, & Orlien, 2019). These enzymes not only assault proteins but also undertake an indispensable catalytic function in the process of protein oxidation. Protein carbonylation is the result of the oxidation described above and is accompanied by changes in the amino acid backbone and side chains. Thus, UHP treatment promoted the oxidation of MP, and the degree of MP oxidation was closely related to the pressure of UHP treatment. According to Shi et al. (2020), when crayfish MP samples were subjected to pressures exceeding 100 MPa, the levels of carbonyl compounds demonstrated a pronounced increase. The authors hinted at a plausible connection between protein oxidation and the functional attributes of muscle-based products, encompassing features like gelation potential and WHC.

### 3.2 EAI and ESI

The EAI measure represents the stability of the interface per unit of protein mass as well as the properties of protein solutions and emulsions composed of oil and water. On the other hand, ESI characterizes the ability of a protein to maintain an emulsion without the separation of oil and water. It was evident from the results depicted in Fig. 1 B-C that the emulsification performance of MP exhibited improvements subsequent to undergoing UHP treatment, as there were significant enhancements observed in both EAI and ESI. The value of EAI recorded an increase from 31.01 m^2^/g to 51.01 m^2^/g (reflecting a percentage increase of approximately 64.50 %) within the UHP pressure range of 0 to 400 MPa. Meanwhile, the value of ESI experienced a surge from 29.02 min to 71.15 min (showing an increase of approximately 1.45-fold). During a study examining the impact of varying UHP levels on *Mytilus edulis* MP, it was observed that as the UHP increased within the range of 20 to 80 MPa, both EAI and ESI showed a gradual rise, ultimately reaching their peak at 80 MPa (Yu et al., 2018). The ability of an emulsion to emulsify depends primarily on the way its components aggregate and the hydrophobic interactions present (Manoi & Rizvi, 2009). Under the influence of UHP, the MP of Tai Lake whitebait displayed a moderate dissociation, which in turn exposed its internal hydrophobic groups. This exposure resulted in reduced tension at the interface between water and oil in the emulsion, ultimately increasing its molecular flexibility and enhancing its emulsification properties. The present study suggested that the emulsifying properties of MP could be improved significantly by UHP treatment (400 MPa for 10 min).

### 3.3 Tertiary conformations

#### 3.3.1 Surface hydrophobicity

**Fig. 2:**
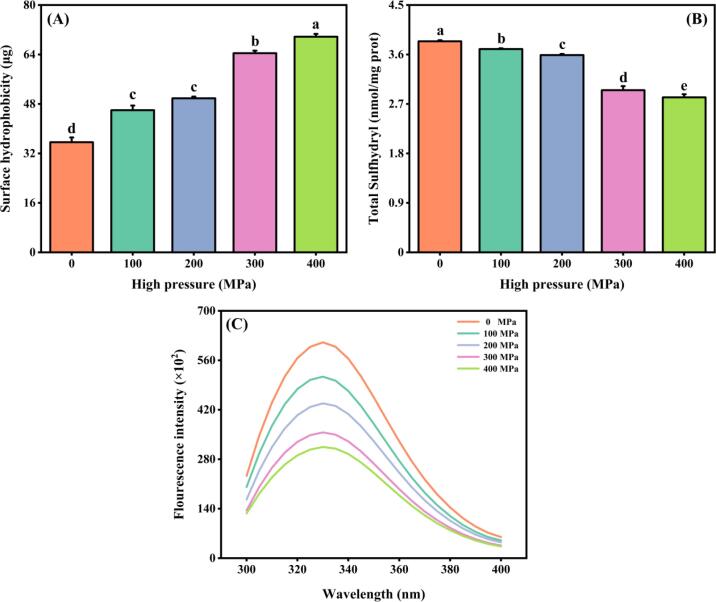
Surface hydrophobicity (A), total sulfhydryl content (B), and endogenous fluorescence spectroscopy (C) of MP samples after UHP treatment. Different lowercase letters on each column represent statistically significant differences (*p* < 0.05).

Fig. 2A demonstrates a notable rise in the hydrophobic characteristic of MP as the UHP intensity increases. The H_0_ of the samples treated with an UHP of 400 MPa was measured at 69.73 μg, which accounted for approximately 95.76 % of the control group. The H_0_ of the protein surface plays a crucial role in the interaction between protein molecules and the surrounding solvent water molecules. Under pressure, the conformation of a protein undergoes denaturation undergoes denaturation, causing previously concealed amino acid residues on the protein surface to be exposed, resulting in an increased H_0_. A study conducted on *Cirrhinus molitorella* has confirmed the existence of a positive correlation between the pressure level and the H_0_, indicative of the enhanced protein unfolding and pronounced exposure of hydrophobic groups induced by pressure (Liu et al., 2022). These findings are consistent with our own investigation. Additionally, it is evident that the H_0_ value has a significant influence on the emulsification of MP. The exposure of hydrophobic groups leads to an increase in the oil retention capacity, which in turn facilitates the binding of MP and oil.

#### 3.3.2 Sulfhydryl group

Sulfhydryl groups, being the most reactive functional group present in MP, have the ability to establish feeble secondary bonds. These bonds are crucial for upholding the protein's tertiary structure and exhibit a significant impact on its stability (Zhang, Yang, Zhou, Zhang, & Wang, 2017). Fig. 2B presents the impact of UHP treatment on the concentration of the overall sulfhydryl compound in Tai Lake whitebait MP. As the UHP intensity rose from 0 to 400 MPa, there was a marked decline in the overall sulfhydryl content, reducing from 3.84 to 2.82 μmol/g (reflecting a 26.56 % decrease). A previous investigation similarly demonstrated a considerable drop in the overall sulfhydryl content of *Patinopecten yessoensis* MP as pressure levels increased (Liu, Mao, Sang, Tian, Ding, & Deng, 2022). The overall sulfhydryl group in the protein consists of both the active sulfhydryl group on its surface and the hidden hydrophobic group within it. The protein conformation of MP was denatured upon exposure to UHP treatment, as evidenced by observed changes and the exposure of internal sulfhydryl groups. The sulfhydryl groups on the protein's surface are prone to oxidation, leading to the formation of disulfide bonds and protein aggregation. This ultimately results in a decline in MP's total sulfhydryl content and is correlated with an increase in H_0_ (Cando, Herranz, Borderias, & Moreno, 2015). Additionally, the outcomes of EAI and ESI exhibited conformity with the H_0_ patterns, arising from the protein aggregation surrounding the oil droplets and yielding a constant, gel-like film.

#### 3.3.3 Endogenous fluorescence intensity

The fluorescence spectrum reflected the tertiary structure of samples of MP. To detect the fluorescence intensity resulting from conformational changes in tryptophan (Trp), phenylalanine (Phe), and lysine (Lys) residues, the excitation wavelength of 280 nm was selected. As shown in Fig. 2C, the maximum fluorescence intensity of the endogenous fluorescence spectrum decreased as the pressure increased from 0 to 400 MPa, suggesting that the protein's tertiary structure was altered by UHP treatment. UHP exerted robust mechanical forces and generated cavitation, exposing Trp, Phe, and Lys residues to an external polar environment, ultimately inducing changes in the protein's conformation. In addition, UHP has the ability to reduce the distance between tryptophan residues and amino acid residues that extinguish fluorescence, consequently leading to a decrease in fluorescence intensity. This outcome resembles the findings from the investigation on the MP of *Patinopecten yessoensis* (Liu, Mao, Sang, Tian, Ding, & Deng, 2022). In contrast, the bond of disulfide has the ability to extinguish the tryptophan residue while it is in an energized state. The fluorescence quenching caused by UHP, which is induced by UHP itself, might occur due to the existence of neighboring disulfide bonds near the tryptophan residues in their energized state. The present study demonstrated that UHP treatment increased the exposure of Trp and Tyr residues to the polar environment on the protein molecule's surface. This suggested that UHP treatment could induce changes in the protein's tertiary structure. Among the treatment groups, the 400 MPa treatment had the most significant effect on the tertiary structure of MP.

### 3.4 Secondary structure of MP

**Fig. 3:**
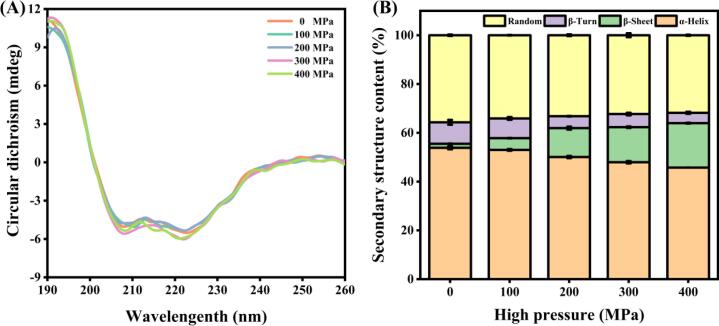
CD spectra (A) and relative content (B) of secondary structure of MP samples after UHP treatment.

To evaluate the secondary structure of Tai Lake whitebait MP, CD spectrometer was employed (Fig. 3A). Notably, the samples treated with UHP exhibited a significant decrease in α-helix content while displaying a substantial increase in β-sheet content when compared to control samples. As depicted in Fig. 3B, the α-helix content of MP subjected to 400 MPa treatment was found to be 45.75 %, signifying a 15.04 % reduction in comparison to the control group (53.85 %). On the other hand, the β-sheet content escalated to 18.20 % in the 400 MPa-treated sample, highlighting a 10.03-fold increase relative to the control group (1.65 %). The proportion of β-turn and random curl remained fairly consistent throughout the experiment. There was a noticeable correlation between the stability of the α-helix structure and the presence of intramolecular hydrogen bonding (Wang, Li, Zheng, Zhang, & Guo, 2019b). Under increasing pressure, the protein underwent unfolding, leading to the weakening of hydrogen bonds. This unfolding process also exposed the previously hidden hydrophobic group in the side chain of amino acids, which facilitated the transition from α-helix to a more loosely arranged β-sheet structure. The decrease in α-helix content and the simultaneous increase in β-sheet formation are commonly associated with unfolding and subsequent aggregation of the MP. A prior study speculates that the MP of red swamp crayfish experiences a gelation process to some extent under UHP conditions, leading to protein aggregation and alterations in secondary structure (Shi et al., 2020).

### 3.5 Rheological properties of MP

**Fig. 4:**
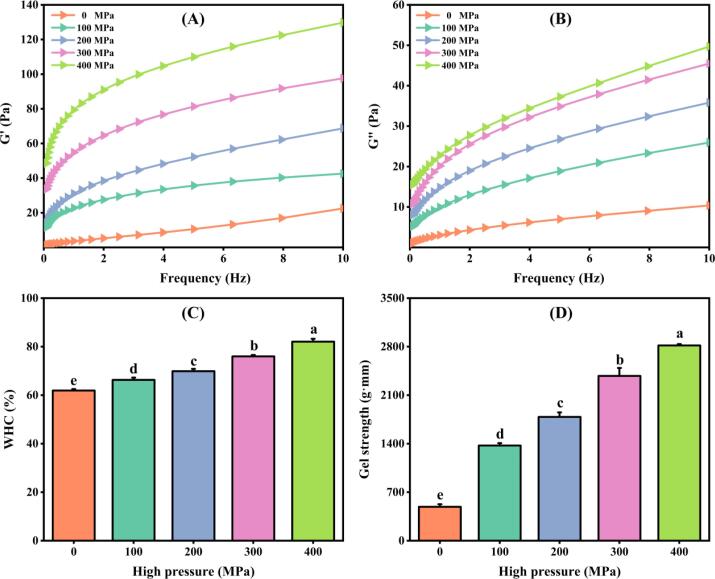
Storage modulus (A), loss modulus (B), gel WHC (C), and gel strength (D) of MP samples after UHP treatment. Different lowercase letters on each column represent statistically significant differences (*p* < 0.05).

The data shown in Fig. 4A-B illustrates the correlation between the values of G' and G“ in Tai Lake whitebait MP under various pressure conditions. The results showed that both G' and G” exhibit a positive trend as the frequency increases in each sample. Notably, the gel's viscoelastic properties were significantly enhanced under higher pressure conditions, with the maximum value being recorded at 400 MPa. This finding suggests that an increased pressure positively influences the structural integrity of the gel network, resulting in improved viscoelastic characteristics (Zhang et al., 2023, Du et al., 2022). In line with the measured sulfhydryl levels and H_0_ (Fig. 2A-B), it presents complete concurrence. UHP treatment results in the production of disulfide connections in MP and heightened hydrophobic associations, thereby facilitating aggregation amid proteins and generating gel formation. This, in turn, amplifies G' and G“.

### 3.6 WHC of MP gel

The texture and sensory characteristics of surimi products are influenced by the gel properties of MP. The formation of MP gels typically involves denaturation and aggregation. Through physical and chemical reactions, the resulting structure of the protein gel network effectively retains water, fat, and other constituents (Zhou, Lin, Liang, Benjakul, Shi, & Liu, 2014). As demonstrated in Fig. 4C, the application of UHP resulted in a remarkable enhancement of MP's WHC. With increasing pressure from 0 to 400 MPa, the WHC exhibited a notable rise from 61.92 % to 82.06 %, representing a substantial increase of 32.53 %. It is widely acknowledged that the WHC of gels is intricately linked to the structure of the cavities and matrix within the network, as well as the protein-water interactions occurring in the gel matrix (Han, Wang, Xu, & Zhou, 2014). The binding of adjacent myosin can be improved by UHP treatment during gelation, which in turn affects the degree of MP unfolding. Additionally, the application of UHP treatment induces protein denaturation, exposing hydrophobic groups and increasing the hydrophobicity within the gel system. As a result, a stronger bond is formed between the protein and water, leading to an increased WHC of MP. Guo, Li, Wang, and Zheng (2019) conducted a study in which they investigated the effects of UHP treatment below 400 MPa on the WHC of golden threadfin bream MP. In addition, the surimi products' texture was enhanced by UHP treatment, which led to the elevation of MP's WHC. A study conducted by Liu et al. (2022) showcased a significant increase in WHC of sausage products made from *C. molitorella* when subjected to higher pressure. Consequently, UHP has proven to enhance MP's WHC across various material sources, exhibiting varying degrees of improvement.

### 3.7 Gel strength

Fig. 4D illustrates the impact of UHP on the gel strength of MP. The application of UHP resulted in a significant increase in gel strength, which was further enhanced as the pressure was gradually increased. Specifically, upon reaching a pressure of 400 MPa, the gel strength exhibited an approximately 4.76-fold increment compared to the control group. The enhanced gel strength can be attributed to the alteration of molecular arrangement within MP gels induced by UHP processing. This process leads to a stronger intermolecular interaction force, resulting in a substantial improvement (Liu et al., 2021). The investigation by Guo et al. (2019) also demonstrated similar findings regarding golden threadfin bream MP. This phenomenon was attributed to the successful alteration of proteins caused by UHP, which in turn facilitated the organized clustering of adjacent protein molecules to form structured gel matrices. The effect of UHP on the gel strength of MP shows significant variation, depending on the specific species being studied.

### 3.8 Microstructure of MP gel

**Fig. 5:**
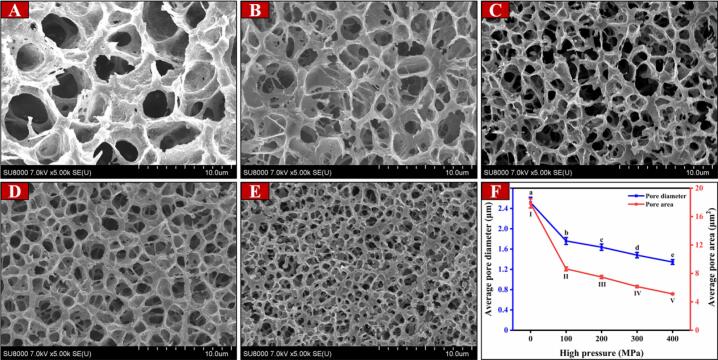
SEM microstructures (A: 0 MPa, B: 100 MPa, C: 200 MPa, D: 300 MPa, E: 400 MPa), and quantitative calculation of microstructures (F) of gels prepared by MP after UHP treatment. Different letters in Fig. 5F represent statistically significant differences (*p* < 0.05).

The functional properties of MPs are greatly influenced by their microstructure, making it an important aspect to consider. Fig. 5A-E visually presents the microstructure of MP gels when subjected to various pressure levels. Moreover, Fig. 5F provide insights into the average pore diameter and average pore area of the different MP gel samples. The untreated MP gel displayed an irregular and non-uniform morphology, which led to weak WHC and gel strength. This can be attributed to its loose and collapsed microstructure. However, with increasing pressure, MP gradually transformed into a compact and homogeneous network with numerous small cavities. Compared to the control group, there was a significant decrease in pore diameter and pore area. According to the research conducted by Liu et al. (2022), it was found that UHP has the capability to compress the microstructure of MP gels within a specific range. This compression effect of UHP resulted in reducing the overall sulfhydryl content within MP, consequently promoting the formation of disulfide bonds among proteins. Ultimately, this led to the development of a favorable network structure during the MP gelation process, thereby improving both WHC and gel strength. Furthermore, Zhang et al. (2017) proposed that this occurrence is linked to the rate at which MP unfolds and aggregates. The application of UHP treatment increases the hydrophobicity and promotes the formation of disulfide bonds, which in turn accelerates the aggregation rate of MP during heating. This results in the development of a compact and uniform framework.

### 3.9 In vitro digestive properties of MP gel

**Fig. 6:**
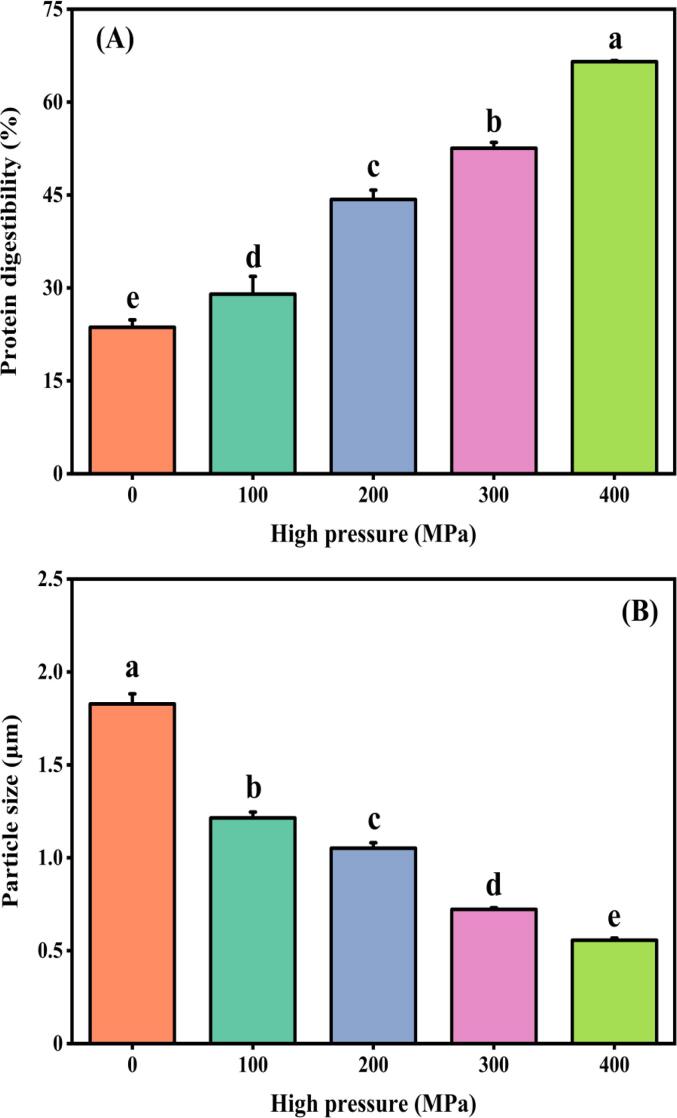
Protein digestibility (A) and particle size (B) of gels prepared by MP after UHP treatment. Different lowercase letters on each column represent statistically significant differences (*p* < 0.05).

Protein digestibility is a crucial factor in evaluating the nutritional quality of food proteins as it provides insights into the extent to which proteins are broken down by digestive enzymes in the digestive tract. Fig. 6A illustrates the variations in *in vitro* digestibility of MP gels under different treatments. Notably, the *in vitro* digestibility of samples treated with UHP was significantly higher, increasing with increasing pressure levels. At 400 MPa, the *in vitro* digestibility of MP gels reached 66.51 %, which was 1.81-fold higher than that of the control group. This phenomenon could be attributed to the structural changes caused by UHP in MP, which leads to partial unfolding and exposes more enzymatic sites. As a result, pepsin and trypsin can bind more easily to MP. In a previous study, it was also confirmed that the amino acids in the side chains of MP were exposed due to the unfolding of MP during UHP treatment. This resulted in the cleavage of specific sites such as Phe, Tyr, and Lys by pepsin or trypsin. Consequently, this led to an increase in the digestibility of MP *in vitro* (Liu, Mao, Sang, Tian, Ding, & Deng, 2022).

After undergoing the hydrolysis process by pepsin and trypsin, the MP gels experienced degradation. This degradation led to the breakdown of high molecular weight proteins into peptides or amino acids with lower molecular mass. As a result, the particle size of the digested product was reduced. As depicted in Fig. 6B, the particle size of the MP gels subjected to various treatments was examined. Notably, the particle size of the sample treated at 400 MPa measured 0.56 μm, which was merely one-third of the control's particle size (1.83 μm). According to a study by Promeyrat et al. (2010), they found a correlation between particle size and the hydrophobicity of proteins. By increasing the hydrophobicity on the surface of MP, it leads to an increased exposure of hydrophobic groups. This process leads to the creation of additional enzymatic cleavage sites, allowing for more efficient hydrolysis of peptide bonds containing hydrophobic amino acid groups within MP by pepsin and trypsin. As a result, MP undergoes a more extensive degradation. This finding is consistent with previous research on the surface hydrophobic effect of UHP and suggests that it can be utilized to improve MP digestion.

## 4 Conclusion

Our observations suggest that UHP significantly affects the physicochemical characteristics, gel properties, and digestibility of Tai Lake whitebait MP. UHP leads to a decrease in the overall content of sulfhydryl groups, while simultaneously enhancing the hydrophobicity of the MP surface. While the composition of the MP remains relatively unaffected as the UHP intensity increases from 0 to 400 MPa, there is an observed increase in the extent of structural aggregation. Furthermore, UHP leads to a significant enhancement in gel strength and WHC of the Tai Lake whitebait MP gels, resulting in a notably denser microstructure. Additionally, UHP improves the *in vitro* digestibility of MP gels and reduces the particle size of digested MP gels. Given the impact of UHP treatment on the physicochemical properties and digestibility of MP, ongoing studies are focusing on the development of digestible Tai Lake whitebait products. The objective of this work is to establish a scientific and theoretical basis for developing Tai Lake whitebait products that are easier to digest and to explore the comprehensive utilization of Tai Lake whitebait meat that is more suitable for children and the elderly.

## CRediT authorship contribution statement

**Mingfeng Xu:** Funding acquisition, Investigation, Methodology, Writing – original draft, Resources. **Xiangxiang Ni:** Methodology. **Qiwei Liu:** Validation. **Chengcheng Chen:** Data curation, Formal analysis. **Xiaohong Deng:** Investigation, Methodology. **Xiu Wang:** Conceptualization, Funding acquisition, Supervision. **Rongrong Yu:** Project administration, Supervision, Writing – review & editing.

## Declaration of competing interest

The authors declare that they have no known competing financial interests or personal relationships that could have appeared to influence the work reported in this paper.

## Data Availability

Data will be made available on request.
