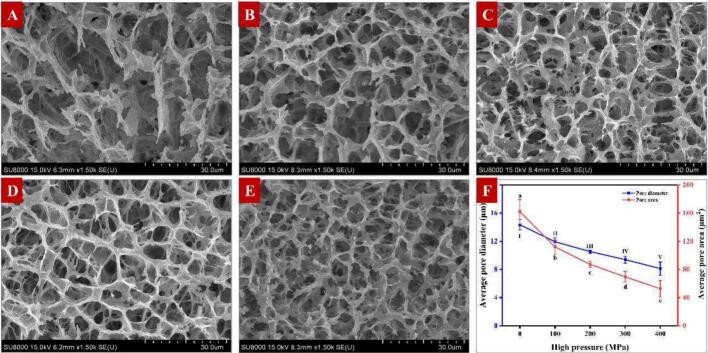# Corrigendum to “Ultra-high pressure improved gelation and digestive properties of tai Lake whitebait myofibrillar protein” [Food Chemistry: X (2024) 101061]

**DOI:** 10.1016/j.fochx.2025.102224

**Published:** 2025-02-07

**Authors:** Mingfeng Xu, Xiangxiang Ni, Qiwei Liu, Chengcheng Chen, Xiaohong Deng, Xiu Wang, Rongrong Yu

**Affiliations:** a College of Life and Environmental Sciences, Hangzhou Normal University, Hangzhou 311121, China; b School of Advanced Materials & Engineering, Jiaxing Nanhu University, Jiaxing 314001, China; c The First Affiliated Hospital of Wenzhou Medical University, Wenzhou 325000, China

The authors regret that the printed version of the above article contained a serious error in Fig. 5. The correct and final version follows. The authors would like to assert that there is no change in the body text of the article. The authors would like to apologise for any inconvenience caused.

Corrigendum

Fig. 5: **(A)**

**Figure:**
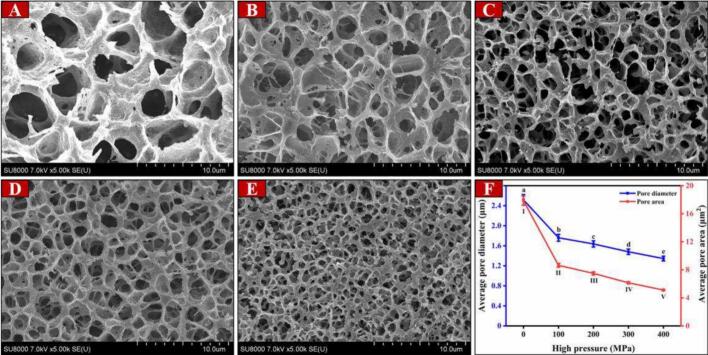

The corrected figure is shown as below **(B)**.

**Figure:**